# Comparing Frailty Status Among Clusters Identified Based on EQ-5D-5L Dimensions in Older Patients with Chronic Low Back Pain

**DOI:** 10.3390/medicina61071217

**Published:** 2025-07-03

**Authors:** Hee Jung Kim, Hyeon Chang Kim, Jisung Hwang, Shin Hyung Kim

**Affiliations:** 1 Department of Anesthesiology and Pain Medicine, Anesthesia and Pain Research Institute, Yonsei University College of Medicine, Seoul 03722, Republic of Korea; heejkim@yuhs.ac (H.J.K.); jisung0425@yuhs.ac (J.H.); 2 Department of Preventive Medicine, Yonsei University College of Medicine, Seoul 03722, Republic of Korea

**Keywords:** cluster analysis, frailty, health-related quality of life, older patients, chronic low back pain

## Abstract

*Background and Objectives*: In the present study, distinct subgroups of older adults with chronic low back pain (LBP) were identified using cluster analysis based on the five dimensions of the EQ-5D-5L. Using detailed profiles of how chronic LBP affects various facets of health-related quality of life (HRQoL), differences in frailty levels across these subgroups were investigated in this study. *Materials and Methods*: This retrospective study included patients ≥ 60 years of age who visited the pain clinic at a tertiary hospital between March 2022 and February 2023. HRQoL was assessed using the EQ-5D-5L, and frailty was evaluated via the Frailty Phenotype Questionnaire. Hierarchical cluster analysis using the WARD method with squared Euclidean distance was conducted on the EQ-5D-5L dimensions to identify subgroups. Differences in frailty, demographics, and clinical data across clusters were analyzed. *Results*: Among 837 older adults with chronic LBP, four distinct clusters were identified based on a cluster analysis of the EQ-5D-5L dimensions. Cluster 1 exhibited high levels of pain/discomfort and anxiety/depression, and cluster 2 had severe mobility limitations and pain/discomfort but low anxiety/depression. Cluster 3 showed balanced scores across all dimensions, and cluster 4 had severe pain/discomfort but good mobility. Significant differences were observed among the clusters in pain intensity, EQ Visual Analogue Scale (EQ-VAS) and EQ-5D-5L index scores, and frailty status. Cluster 1 had the highest pain scores and lowest EQ-VAS, and frailty was most prevalent in cluster 2 (28.5%) and least in cluster 4 (13.3%). *Conclusions*: The results of the present study emphasize the complexity of chronic LBP in older adults by identifying distinct clusters. Cluster analysis identified four unique profiles, with significant frailty differences across the clusters. These findings emphasize the importance of personalized management strategies tailored to specific patient profiles to enhance treatment effectiveness and improve frailty status.

## 1. Introduction

Low back pain (LBP) is a prevalent issue affecting all age groups globally and has become the leading cause of disability worldwide [1]. A significant proportion of patients develop chronic LBP, affecting approximately 23% of individuals and severely affecting their functional ability and quality of life [2,3,4]. Older adults with chronic LBP often experience exacerbated psychosocial dysfunction, increased self-reported disability, and reduced physical capacity [5].

Health-related quality of life (HRQoL) is a multifaceted concept that encompasses an individual’s functioning and perceived well-being across physical, mental, and social aspects [6]. The HRQoL is used to evaluate how physical limitations, pain, and discomfort affect daily activities, social interactions, and psychological health [7]. The EQ-5D-5L is a crucial tool for measuring HRQoL and assesses five specific dimensions: mobility, self-care, usual activities, pain/discomfort, and anxiety/depression [8]. Responses to such instruments can vary widely among patients with chronic LBP, reflecting the complex interplay of biophysical, psychological, and social factors that affect individuals differently [1]. Thus, identifying homogenous subgroups using machine learning algorithms is crucial for tailoring treatment and improving care quality [9].

Frailty, a critical concept in geriatrics, is defined as a clinical syndrome characterized by reduced physiological reserves and increased vulnerability to stressors, often manifesting as weakness, fatigue, and unintentional weight loss [10]. This condition significantly increases the risk of adverse health outcomes in older adults [11]. A strong association between frailty and HRQoL has been highlighted in recent studies [12,13], with previous research [14] further demonstrating this relationship in older adults with chronic LBP.

Comparing frailty within clusters based on specific HRQoL dimensions is essential for understanding how various aspects of a patient’s life affect their frailty status. Classifying chronic LBP patients using the EQ-5D-5L helps identify distinct subgroups with varying physical and psychological profiles. When comparing frailty across these clusters, how specific HRQoL dimensions, such as mobility limitations, severe pain, or high anxiety, correlate with frailty can be determined. Understanding how frailty differs across various HRQoL profiles will provide important insights into personalized treatment strategies and interventions. This comparison enhances research by reducing heterogeneity and improving treatment efficacy and leads to more targeted and effective patient care [15].

In the present study, cluster analysis was performed on older patients with chronic LBP based on the five dimensions of the EQ-5D-5L to identify distinct subgroups. Subsequently, differences in frailty status among the subgroups were investigated.

## 2. Materials and Methods

### 2.1. Ethics and Consent

This retrospective cross-sectional study was reviewed and approved by the Institutional Review Board of Yonsei University Health System, Seoul, Republic of Korea (approval number 4-2024-0727, dated 1 August 2024). Due to the retrospective and observational nature of this study, the Institutional Review Board waived the requirement for obtaining informed consent from patients.

### 2.2. Study Population

The patients enrolled in this study were subjects who first visited the pain clinic at Severance Hospital, a leading tertiary hospital in Korea, between March 2022 and February 2023. Patients ≥ 60 years of age with complaints of LBP were included in the study. Patients with acute pain within the past 3 months and subjects with incomplete electronic medical records were excluded.

### 2.3. Evaluation of HRQoL

HRQoL was assessed at the pain clinic during the first patient visit using the EQ-5D-5L tool. Created by the EuroQol Group, the EQ-5D-5L is accessible in 102 languages on the EuroQol website (http://www.euroqol.org (accessed on 1 July 2025)) and consists of two parts: the EQ-5D descriptive system and the EQ Visual Analogue Scale (EQ-VAS).

The EQ-5D descriptive system comprises five dimensions: mobility, self-care, usual activities, pain/discomfort, and anxiety/depression. Each dimension has five levels ranging from no problem to an extreme problem. The EQ-VAS allows individuals to assess their overall health on a scale from 0 to 100, where 0 signifies the worst possible health and 100 represents the best possible health. In addition, social index values for EQ-5D-5L health states among the Korean population were established using the composite time trade-off method, as outlined in a prior study [16].

### 2.4. Frailty Screening

Frailty screening was performed during the first patient visit to the pain clinic for pain management. Frailty was assessed using the Frailty Phenotype Questionnaire [17] which includes five components:

1. Fatigue: Participants receive 1 point if they report feeling that everything they did was a lot of effort for more than 3 days a week in the past week, otherwise 0 points.
2. Resistance: Participants receive 1 point if they have difficulty climbing 10 stairs without assistance, otherwise 0 points.
3. Ambulation: Participants receive 1 point if they report any difficulty walking around a 400 m playground track, otherwise 0 points.
4. Inactivity: Participants receive 1 point if they do not engage in moderate or vigorous physical activity in the past 7 days, otherwise 0 points.
5. Unintentional weight loss: Participants receive 1 point if they lost 4.5 kg or more unintentionally in the past year, otherwise 0 points.

Based on the Fried frailty phenotype assessment, individuals scoring 0 were classified as robust, 1–2 as pre-frail, and 3 or higher as frail.

### 2.5. Patient Demographics and Clinical Data Measurements

Patient demographics and pain-related data were collected from electronic medical records during the initial outpatient clinic visit. Demographic information included age, sex, body mass index (BMI), diagnosed comorbidities (hypertension, diabetes mellitus, cancer, mental health disorders, and osteopenia/osteoporosis), and previous spinal surgery history. In addition, the pain-related data collected included baseline pain scores using the Numeric Rating Scale (NRS), pain duration, opioid use for ≥1 month prior to the visit, and the presence of sleep disturbances. Pain severity was categorized based on NRS scores as follows: mild (1–3), moderate (4–6), or severe (7–10) [18].

### 2.6. Statistical Analysis

Continuous variables were presented as mean ± standard deviation (SD), and categorical variables were tabulated as numbers (percentage). The normality of distribution was assessed using the Shapiro–Wilk test. Differences among the cluster groups were analyzed using the one-way analysis of variance, Chi-squared, and Kruskal–Wallis tests. Bonferroni’s multiple comparison or Chi-squared post hoc tests were used to determine intergroup differences when significant differences were found. Statistical Package for the Social Sciences, version 28.0 (IBM Corp, Armonk, NY, USA), was used for data analysis. A *p*-value < 0.05 was considered statistically significant.

### 2.7. Cluster Analysis

Hierarchical cluster analysis was conducted to subgroup patients based on the relevance of the EQ-5D-5L dimensions. To standardize individual perceptions, an alternative score was calculated by subtracting the average value of each of the five dimensions. In this alternative scoring method, values > 0 indicate more pronounced problems compared to the individual average perception and values < 0 indicate less intense problems.

The hierarchical WARD method with squared Euclidean distance was utilized for clustering. This method was chosen due to its ability to minimize within-cluster variance and its suitability for exploratory subgroup identification, especially when combined with dendrogram-based visual inspection. The analysis was performed using Python version 3.8.15 with Scikit-learn version 1.0.2. Essential clusters were defined using a cut-off point of approximately 10% of the total cases. A four-cluster solution was selected to maintain the significant differences identified using agglomerative clustering. Hierarchical clustering resulted in four distinct clusters that met our predefined criteria.

Validation and case reorganization were conducted using k-means cluster analysis, confirming consistent results. Although EQ-5D-5L dimensions are ordinal, we applied within-individual centering and standardization to treat the adjusted scores as continuous variables, justifying the use of squared Euclidean distance. EQ-VAS was not included in the clustering algorithm but was used post hoc to compare health status across clusters.

## 3. Results

**Figure 1:**
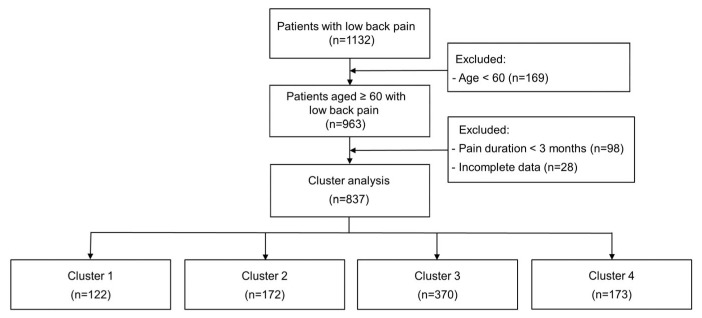
Study flowchart.

From March 2022 to February 2023, among the 1132 LBP patients who visited the pain clinic, 837 patients ≥ 60 years of age with chronic LBP were included in the study after excluding subjects who met the exclusion criteria (Figure 1).

The demographic profiles and pain-related data of the enrolled participants are detailed in Table 1. The mean age of the patients was 72.01 years, with females comprising over 50% of the group. The median BMI was 22.89 kg/m^2^. Hypertension was reported in 251 patients, diabetes mellitus in 146, cancer in 130, mental health problems in 107, and osteopenia/osteoporosis in 104. In addition, 197 patients (23.5%) had a history of spine surgery. A total of 5.5% of patients reported mild pain (NRS 1–3), 39.3% reported moderate pain (NRS 4–6), and 55.2% reported severe pain (NRS 7–10). The median duration of pain was 12 months. Approximately 30% of patients reported using opioids and experiencing sleep disturbances. The most commonly reported EQ-5D-5L dimension at levels > 2 was pain/discomfort (70.1%), followed by mobility (51.8%), usual activities (29.6%), anxiety/depression (25.6%), and self-care (17.2%). The mean EQ-VAS score was 61.87 and the mean EQ-5D-5L index score was 0.63. The frailty status of the patients included 244 (29.2%) classified as robust, 409 (48.9%) as pre-frail, and 184 (22.0%) as frail.

**Figure 2:**
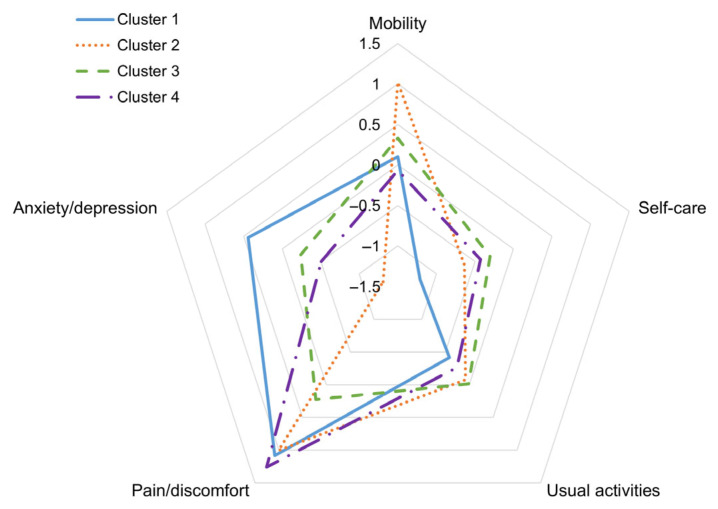
The distribution of the EQ-5D-5L dimensions across the four cluster groups.

A cluster analysis was conducted using the EQ-5D-5L questionnaire to subgroup patients based on the relevance of the dimensions, resulting in four distinct clusters (Figure 2). Cluster 1 included patients with high levels of pain/discomfort and anxiety/depression but generally good self-care abilities. Cluster 2 consisted of patients who experienced severe mobility limitations and pain/discomfort but exhibited low levels of anxiety/depression. Cluster 3 included patients with similar scores across all dimensions, indicating a generally balanced condition without extreme issues in any particular area. Cluster 4 consisted of patients suffering from severe pain/discomfort but who maintained good mobility and relatively low levels of anxiety/depression.

A comparison was conducted to examine the differences in patient characteristics and pain-related data among the four clusters (Table 2). No significant differences were found in age, BMI, and comorbid medical conditions across the clusters. However, a significant difference was found in the proportion of female patients, with cluster 2 having the lowest proportion (57.0%) compared to cluster 1 (70.5%), cluster 3 (69.7%), and cluster 4 (69.4%) (*p* = 0.017).

Severity distribution differed significantly, with clusters 1 and 2 showing higher proportions of severe pain and lower proportions of mild pain compared to clusters 3 and 4 (*p* < 0.001). A trend towards longer pain duration was observed in cluster 3 (median: 24 months) but without statistical significance (*p* = 0.050). No significant differences were observed in opioid usage and sleep disturbance among the clusters.

**Figure 3:**
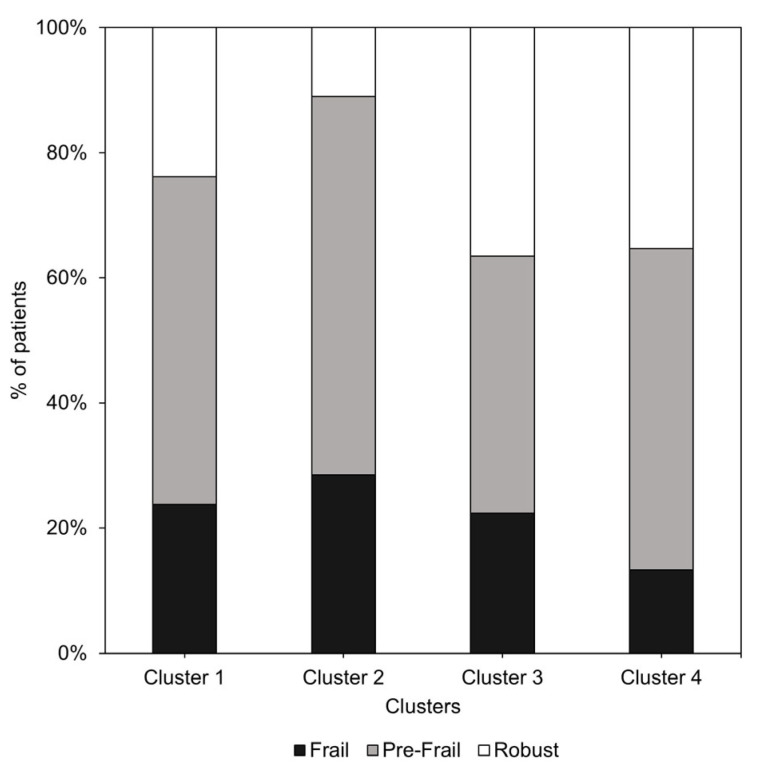
The distribution of frailty status categorized based on the four clusters.

Patients in cluster 1 reported significantly lower EQ-VAS scores (56.80 ± 18.53) compared to the other clusters (*p* = 0.005). Furthermore, clusters 1 and 2 had a lower EQ-5D-5L index (0.55 ± 0.18 and 0.55 ± 0.17, respectively) compared to clusters 3 and 4, indicating a poorer overall health status (*p* < 0.001). In addition, the proportion of frail patients was highest in cluster 2 (28.5%) and lowest in cluster 4 (13.3%; *p* < 0.001; Figure 3).

## 4. Discussion

In the present study, homogeneous subgroups were identified based on the five dimensions of the EQ-5D-5L among older patients with chronic LBP. In addition, differences in frailty status and other relevant factors within the subgroups were examined. In the study, four distinct clusters of these patients were identified based on their EQ-5D-5L dimensions and significant differences in frailty status were observed.

Patients in cluster 1 experienced high levels of pain/discomfort and anxiety/depression and had lower EQ-VAS scores and elevated frailty, indicating that significant mental strain is associated with increased frailty. Cluster 2 was characterized by severe mobility limitations and significant pain/discomfort and exhibited the highest levels of frailty, indicating a close relationship between severe mobility limitations, high pain scores, and increased frailty. In contrast, patients in cluster 4, despite experiencing severe pain and discomfort, maintained good mobility and reported lower levels of anxiety and depression. In addition, cluster 4 had the lowest level of frailty among all clusters. Patients in cluster 3 had balanced scores across all EQ-5D-5L dimensions, indicating a relatively stable condition and higher overall health. In cluster 3, maintaining current health through pain management and preventive care was important to avoid the further progression of frailty.

Due to the multifactorial nature of chronic LBP, various approaches to identify subgroups of patients have been examined in previous studies [19,20,21,22]. Results have shown that tailoring treatments to specific subgroups based on their clinical presentations can lead to improved clinical outcomes, increased overall health benefits, and cost savings [21,22]. The research in the present study extends this work by examining the association between frailty and the subgroups identified using cluster analysis of the EQ-5D-5L dimensions. Frailty is a multidimensional and dynamic condition influenced by both physical and psychosocial factors [10]. In a systematic review regarding transitions between frailty states, approximately 43% of individuals were shown to experience at least one transition, encompassing both deterioration and improvement [23]. Therefore, implementing personalized interventions tailored to subgroups identified based on cluster analysis can modify the frailty status by enhancing the independence, physical functioning, and cognitive health of individuals [24].

In the present study, severe mobility limitations, as observed in cluster 2, appeared to be closely associated with the highest levels of frailty. More than 50% of patients with chronic severe LBP experience some form of mobility or work impairment [25]. In addition, Coyle et al. [26] showed that overall frailty was significantly more prevalent in older adults with chronic LBP than in subjects without pain. These findings reinforce the role of chronic LBP in contributing to mobility limitations and frailty and emphasize the importance of physical activity interventions for patients with mobility issues [27,28]. Psychological distress, such as anxiety and depression, plays a critical role in frailty and was particularly evident in cluster 1. Both pre-frail and frail elderly individuals experienced higher levels of anxiety and depression compared to their robust counterparts [29]. Furthermore, an association between depressive symptoms and the development of frailty was reported in a large epidemiological study [30]. Therefore, effective management of frailty in this subgroup experiencing psychological distress necessitates addressing psychosocial factors, including social support and coping strategies. Chronic pain often impairs mobility, leading to social isolation and increased emotional distress [31]. However, as observed in cluster 4, despite experiencing severe chronic pain, individuals who maintained good mobility and low levels of psychological distress experienced a relatively low level of frailty. This emphasizes the interrelated nature of physical activity and psychological distress [32,33] and demonstrates that promoting both physical activity and psychological recovery can improve frailty status in elderly patients with chronic LBP [34,35]. Examining frailty in relation to these clusters revealed the complex, multifaceted nature of chronic LBP and provided new insights into how different health dimensions interact with frailty. This novel approach emphasizes the within-person differences in frailty among patients with chronic LBP and shows that more targeted and potentially effective management strategies are needed.

Furthermore, demographic differences, particularly between sexes, were observed across clusters. In cluster 2 in which severe pain and discomfort were reported, the proportion of females was relatively low. This is in contrast with the typically higher pain levels and more intense chronic pain experienced by female patients due to hormonal, psychological, and social factors [36]. The discrepancy may be attributed to lower serum testosterone levels in elderly males, which can contribute to limitations in physical function and mobility [37]. These findings emphasize the complex role of sex in pain experience and the need for a multidimensional pain assessment approach. Further research is necessary to explore these differences more comprehensively.

In addition, although the clusters differed in HRQoL profiles and frailty levels, no significant differences were observed in opioid use or sleep disturbance. This suggests that these outcomes may be influenced by external factors such as prescribing behaviour, coping mechanisms, or comorbid conditions not fully captured by EQ-5D-5L dimensions. Further investigation is needed to clarify these interactions.

The present study had several limitations that should be considered when interpreting the findings. First, the sample was limited to Korean older adults from a specific healthcare setting which may affect the generalizability of the results. Selection bias and lack of diversity could influence the findings. However, the methodology and outcome measures used in this study are widely applicable and may serve as a reference for similar investigations in other populations. Second, the retrospective design relies on existing records, potentially leading to missing data and limiting causal inferences. Third, the EQ-5D-5L and Frailty Phenotype Questionnaire, although useful, may not capture all aspects of chronic LBP and HRQoL, and are subject to self-report bias. In addition, detailed analyses of lumbar pathology or radiological severity, which may affect symptom interpretation, are not included. However, such discrepancies between symptoms and lumbar pathology or radiological severity are common [38] and do not necessarily affect the findings.

## 5. Conclusions

In conclusion, the unique patterns identified using cluster analysis emphasized the complexity of chronic LBP in older adults. Chronic LBP affects physical, mental, and social aspects of life in various ways, as confirmed in the results of the present study showing significant variations in frailty levels across different subgroups. These differences emphasize the need for personalized management strategies, indicating that tailoring treatments to specific patient profiles could enhance intervention effectiveness and improve frailty status. The outcomes of various treatment options in these patient clusters should be investigated in future studies to determine their effectiveness in improving frailty, as also suggested in preliminary findings presented previously [39].

## Figures and Tables

**Table 1:** Patient characteristics, pain-related data, and frailty status of 837 patients with chronic LBP.

Variables	Total (n = 837)
**Patient characteristics**	
Age, years	72.01 ± 7.14 (60–96)
Female sex, n	562 (67.1)
BMI, kg/m^2^	22.89 (20.81; 25.18)
Comorbid medical disease, n	
Hypertension	251 (30.0)
Diabetes mellitus	146 (17.4)
Cancer	130 (15.5)
Mental health problems	107 (12.8)
Osteopenia/osteoporosis	104 (12.4)
Spine surgery history, n	197 (23.5)
**Pain-related data**	
Baseline pain score	
Mild (NRS 1–3), n	46 (5.5)
Moderate (NRS 4–6), n	329 (39.3)
Severe (NRS 7–10), n	462 (55.2)
Pain duration, months	12.00 (6.00; 49.00)
Opioid usage, n	284 (33.9)
Sleep disturbance, n	273 (32.6)
**EQ-5D-5L**	
Mobility, >2, n	433 (51.8)
Self-care, >2, n	144 (17.2)
Usual activities, >2, n	248 (29.6)
Pain/discomfort, >2, n	587 (70.1)
Anxiety/depression, >2, n	214 (25.6)
EQ-VAS, 0–100	61.87 ± 17.89 (10–100)
EQ-5D-5L index	0.63 ± 0.18 (−0.08–1.00)
**Frailty status, n**	
Robust	244 (29.2)
Pre-frail	409 (48.9)
Frail	184 (22.0)

Values are presented as mean ± standard deviation (SD) or number of patients (%). LBP, lower back pain; BMI, body mass index; NRS, Numeric Rating Scale; EQ-VAS, EQ Visual Analogue Scale.

**Table 2:** Characteristic comparison of derived cluster groups.

Cluster Label	Cluster 1 (n = 122)	Cluster 2 (n = 172)	Cluster 3 (*n* = 370)	Cluster 4 (n = 173)	*p*-Value
**Patient characteristics**					
Age, years	71.90 ± 6.69 (60–88)	71.86 ± 7.37 (60–91)	72.13 ± 7.21 (60–96)	71.97 ± 7.12 (60–91)	0.975
Female sex, n **	86 (70.5) ^2^	98 (57.0) ^1,3,4^	258 (69.7) ^2^	120 (69.4) ^2^	0.017
BMI, kg/m^2^	22.62 (20.75; 24.44)	23.14 (21.64; 25.35)	22.89 (20.77; 25.58)	22.83 (20.52; 24.85)	0.141
Comorbid medical disease, n					
Hypertension	37 (30.3)	57 (33.1)	106 (28.6)	51 (29.5)	0.763
Diabetes mellitus	15 (12.3)	35 (20.3)	64 (17.3)	32 (18.5)	0.335
Cancer	19 (15.6)	29 (16.9)	54 (14.6)	28 (16.2)	0.911
Mental health problems	11 (9.0)	22 (12.8)	49 (13.2)	25 (14.5)	0.561
Osteopenia/osteoporosis	13 (10.7)	23 (13.4)	42 (11.4)	26 (15.0)	0.580
Spine surgery history	21 (17.2)	38 (22.1)	96 (25.9)	42 (24.3)	0.245
**Pain-related data**					
Baseline pain score					
Mild (NRS 1–3), n **	5 (4.1) ^3,4^	4 (2.3) ^3.4^	26 (7.0) ^1,2^	11 (6.4) ^1,2^	<0.001
Moderate (NRS 4–6), n	34 (27.9) ^3,4^	54 (31.4) ^3,4^	162 (43.8) ^1,2^	79 (45.7) ^1,2^	
Severe (NRS 7–10), n	83 (68.0) ^3,4^	114 (66.3) ^3,4^	182 (49.2) ^1,2^	83 (48.0) ^1,2^	
Pain duration, months	13.50 (6.00; 60.00)	12.00 (5.00; 36.00)	24.00 (6.00; 60.00)	12.00 (6.00; 38.00)	0.050
Opioid usage, n	38 (31.1)	63 (36.6)	123 (33.2)	60 (34.7)	0.777
Sleep disturbance, n	51 (41.8)	61 (35.5)	112 (30.3)	49 (28.3)	0.053
**EQ-5D-5L**					
EQ-VAS, 0–100 *	56.80 ± 18.53 (10–90) ^2,3,4^	63.84 ± 16.16 (20–100) ^1^	62.01 ± 18.10 (10–100) ^1^	63.21 ± 18.11 (10–100) ^1^	0.005
EQ-5D-5L index *	0.55 ± 0.18 (0.16–0.83) ^3,4^	0.55 ± 0.17 (0.08–0.80) ^3,4^	0.66 ± 0.17 (−0.08–1.00) ^1,2^	0.69 ± 0.15 (0.19–0.86) ^1,2^	<0.001
**Frailty status, n** **					
Robust	29 (23.8) ^2,3,4^	19 (11.0) ^1,3,4^	135 (36.5) ^1,2,4^	61 (35.3) ^1,2,3^	<0.001
Pre-frail	64 (52.5) ^2,3,4^	104 (60.5) ^1,3,4^	152 (41.1) ^1,2,4^	89 (51.4) ^1,2,3^	
Frail	29 (23.8) ^2,3,4^	49 (28.5) ^1,3,4^	83 (22.4) ^1,2,4^	23 (13.3) ^1,2,3^	

Values are presented as mean ± standard deviation (SD) or number of patients (%). BMI, body mass index; NRS, Numeric Rating Scale; EQ-VAS, EQ Visual Analogue Scale. * Bonferroni’s multiple comparison test (*p* < 0.05); ** Chi-square post hoc difference (*p* < 0.05). ^1^, vs. cluster 1; ^2^, vs. cluster 2; ^3^, vs. cluster 3; ^4^, vs. cluster 4.

## Data Availability

The minimal dataset supporting the findings of this study is provided as Supplementary Material to the article. The full dataset contains personal health information and cannot be shared publicly due to institutional and legal restrictions. Data are available from the corresponding author upon reasonable request and with appropriate ethical approval.

## Supplementary Materials

The following supporting information can be downloaded at: https://www.mdpi.com/article/10.3390/medicina61071217/s1.

## Abbreviations

The following abbreviations are used in this manuscript:

LBP	Low back pain
HRQoL	Health-related quality of life
EQ-VAS	EQ Visual Analogue Scale
BMI	Body mass index
NRS	Numeric Rating Scale
SD	Standard deviation
